# GBE1 Is an Independent Prognostic Marker and Associated With CD163^+^ Tumor-Associated Macrophage Infiltration in Lung Adenocarcinoma

**DOI:** 10.3389/fonc.2021.781344

**Published:** 2022-01-27

**Authors:** Yicheng Liang, Yangyang Lei, Mei Liang, Minjun Du, Zixu Liu, Xingkai Li, Xiangzhi Meng, Boxuan Zhou, Yushun Gao

**Affiliations:** ^1^ Department of Thoracic Surgery, National Cancer Center/National Clinical Research Center for Cancer/Cancer Hospital, Chinese Academy of Medical Sciences and Peking Union Medical College, Beijing, China; ^2^ Department of Interventional Radiology, Shanghai Institute of Medical Imaging, Shanghai, China; ^3^ Department of Interventional Radiology, Zhongshan Hospital, Fudan University, Shanghai, China

**Keywords:** clinical characteristics, GBE1, LUAD, CD163+ tumor-associated macrophage infiltration, tissue microarray (TMA), prognosis, immunohistochemistry (IHC)

## Abstract

Glycogen branching enzyme (GBE1) is a critical gene that participates in regulating glycogen metabolism. However, the correlations between GBE1 expression and the prognosis and tumor-associated macrophages in lung adenocarcinoma (LUAD) also remain unclear. Herein, we firstly analyzed the expression level of GBE1 in LUAD tissues and adjacent lung tissues *via* The Cancer Genome Atlas (TCGA) database. The effect of GBE1 on prognosis was estimated by utilizing TCGA database and the PrognoScan database. The relationships between the clinical characteristics and GBE1 expression were evaluated *via* TCGA database. We then investigated the relationships between GBE1 and infiltration of immune cells in LUAD by utilizing the CIBERSORT algorithm and Tumor Immune Estimation Resource (TIMER) database. In addition, we used a tissue microarray (TMA) containing 92 LUAD tissues and 88 adjacent lung tissues with immunohistochemistry staining to verify the association between GBE1 expression and clinical characteristics, as well as the immune cell infiltrations. We found the expression level of GBE1 was significantly higher in LUAD tissues. High expression of GBE1 was associated with poorer overall survival (OS) in LUAD. In addition, high expression of GBE1 was correlated with advanced T classification, N classification, M classification, TNM stage, and lower grade. Moreover, GBE1 was positively correlated with infiltrating levels of CD163^+^ tumor-associated macrophages in LUAD. In conclusion, the expression of GBE1 is associated with the prognosis and CD163^+^ tumor-associated macrophage infiltration in LUAD, suggesting that it has potential to be prognostic and immunological biomarkers in LUAD.

## Introduction

Glycogen metabolism is an important part of the metabolic adaptation mechanisms used by cancer cells to adapt to the tumor microenvironment (1, 2). Glycogen branching enzyme (GBE1) is one of the crucial enzymes in glycogen metabolism, which could catalyze the transfer of alpha-1,4-linked glucosyl units from the outer end of a glycogen chain to an alpha-1,6 position on the same or a neighboring glycogen chain, then consequently, increasing the solubility of the glycogen molecule and reducing the osmotic pressure within cells (3, 4). Previous studies demonstrated that hypoxia-induced GBE1 expression could promote tumor progression through metabolic reprogramming in lung adenocarcinoma (LUAD) (5). Additionally, GBE1 blockade could promote the secretion of CCL5 and CXCL10 to recruit CD8^+^ T lymphocytes to the tumor microenvironment *via* the IFN-I/STING signaling pathway, accompanied by upregulation of PD-L1 in LUAD cells (6). However, the roles of GBE1 in LUAD progression and tumor microenvironment are still needed to further explore.

Accumulating evidence showed that tumor microenvironment and immune-related mechanism played crucial roles in the development and progression as well as treatment of LUAD (7–10). As an important part of tumor microenvironment, tumor-associated macrophages (TAMs) have been reported that they could affect the prognosis and efficacy of chemotherapy and immunotherapy (11–17). Tumor microenvironment could promote monocyte differentiation into M2 TAMs *via* a complex cytokine-based connection, which promotes tumor migration and metastasis (18, 19). Previous studies demonstrated that M0 and M2 macrophages, as well as resting memory CD4^+^ T cells, accounted for the majority of tumor-infiltrating immune cells in LUAD patients (20). CD163 is a scavenger receptor for the hemoglobin-haptoglobin complex and is deemed as a phenotypic marker of anti-inflammatory M2 macrophages (21). It was reported that CD163^+^ tumor-associated macrophages could inhibit T-cell proliferation and activation by secreting CCL22, IL-10, and TGF-β and recruiting regulatory T cells (Tregs) to tumor tissues (22, 23). The tumor islet-infiltrating CD163^+^ tumor-associated macrophages were associated with the prognosis of nonsmall-cell lung cancer (NSCLC) patients (22). Therefore, further analysis of the interaction between M2 TAMs and LUAD is urgently needed.

In this study, the expression level of GBE1 and its prognostic role in LUAD were comprehensively analyzed by utilizing the PrognoScan database, TCGA database and the tissue microarray (TMA)‐based immunohistochemistry (IHC). Moreover, we explored the correlation between GBE1 expression and the tumor-infiltrating immune cells in different tumor microenvironments *via* the CIBERSORT algorithm and TIMER database. Furthermore, we verified the relationship between GBE1 expression and CD163^+^ tumor-associated macrophages *via* IHC staining. Our study may provide new insights into the important roles of GBE1 in LUAD and reveal a potential relationship between GBE1 and tumor microenvironment.

## Material and Methods

### Tissue Microarray and Immunohistochemistry Staining

To explore the expressions of GBE1 and CD163 in LUAD tissues and paired adjacent lung tissues, tissue microarray (HLugA180Su04) containing 92 LUAD tissues and 88 adjacent lung tissues were obtained from Shanghai Outdo Biotech Co, Ltd. All included patients did not receive neoadjuvant therapy and received operation between January 2008 and December 2013. Eliminating 11 ineffective LUAD tissues, a total of 81 LUAD cases included in these two studies. The sections were mounted onto slides coated with 3‐aminopropyltriethoxysilane. After drying and dewaxing, the sections underwent high-pressure antigen retrieval and reacted with antibodies against GBE1 (ab223799, dilution 1:300; Abcam, San Francisco, CA, USA) and CD163 (ab182422, dilution 1:500; Abcam, San Francisco, CA, USA). Furthermore, the slide was incubated with HRP-labeled anti-rabbit IgG (Dako, Glostrup, Denmark) for 45 min at room temperature. Finally, the slide was stained with 3,3-diaminobenzidine (DAB) and was counterstained with hematoxylin. The EnVision+ detection system (Dako) was used according to the manufacturer’s instructions. The score of IHC was calculated by multiplying the intensity (0–3) and extent (0%–100%) of staining for each tissue point. These tissue samples were collected with bioethics approvals and informed consents. The diagnosis of LUAD was validated by pathological evidence, and the tumor grades and clinical stages were classified using the 7th American Joint Committee on Cancer (AJCC) TNM criteria. The experiments were approved by the Ethics Committee of National Cancer Center/Cancer Hospital) Chinese Academy of Medical Sciences and Peking Union Medical College (IRB Approval No. NCC2019C-167).

### Survival Analysis by PrognoScan Database, TCGA Database, and TMA cohort

The correlation between GBE1 expression and prognosis in LUAD was analyzed by the PrognoScan database (http://www.abren.net/PrognoScan/), which collects plenty of publicly available cancer microarray datasets, and consequently, could be utilized to explore the association between a gene and clinical outcome in cancer research (24). The hazard ratio (HR) and adjusted *p*-value were assessed. Furthermore, we explored the correlation between GBE1 expression and prognosis by using TCGA cohort and TMA cohort. The patients with other malignancies were excluded in TCGA cohort. The cutoff values of high and low expression were calculated by R package “OptimalCutpoints.” The Kaplan-Meier method and Log-rank test were used to compare the prognosis of patients with different GBE1 expression. The multivariable Cox regression analysis was used to confirm whether GBE1 is an independent prognostic factor.

### The Relationship Between Clinical Characteristics and GBE1 Expression in LUAD

We further explored the relationship between clinical characteristics and GBE1 expression by using TCGA database and TMA cohort. Level 3 RNA-Seq V2 data for 20,530 genes of lung adenocarcinoma patients (TCGA-LUAD Cohort) were downloaded from the UCSC Xena browser (http://xena.ucsc.edu/) TCGA hub. Updated clinical and survival information for TCGA patients were also obtained from the UCSC Xena browser. The differential expression of GBE1 between paired tumor tissues and normal tissues were analyzed. The age, gender, T stage, N stage, M stage, and TNM stage of LUAD patients were extracted from the TCGA cohort to analyze the GBE1 expression in these different subgroups. In TMA cohort, gender, age, T, N, TNM, and grade were also collected and analyzed. Furthermore, we evaluated the expression of CD163 in the TMA cohort.

### Evaluation of Immune Cell Infiltration

We evaluated the proportions of immune cells in LUAD by using the CIBERSORT method. The CIBERSORT algorithm is an immune cell infiltration estimation analysis tool, which can be used to estimate the immune cell infiltration by deconvoluting the expression matrix of immune cell subtypes based on linear support vector regression (25). Immune cells fraction data downloaded from TCGA Data Portal (https://gdc.cancer.gov/about-data/publications/panimmune, filename: TCGA.Kallisto.fullIDs.cibersort.relative.tsv) (26). Correlation analyses between the expression of GBE1 and immune cell filtration were performed with Pearson’s correlation by using the “corrplot” function with R package corrplot. Furthermore, we also validated the associations between GBE1 expression and correlated immune infiltrates *via* the TIMER database, including B cells, CD4^+^ T cells, CD8^+^ T cells, macrophages, and dendritic cells. TIMER (https://cistrome.shinyapps.io/timer/) is a web server designed for exploring the associations between immune infiltrates and different factors, including gene expression, clinical outcomes, somatic mutations, etc. In total, 10,897 tumors from 32 cancer types were collected to estimate the abundance of immune infiltrates (27). For further investigation, the correlations of GBE1 expression with gene markers of M1 macrophage (NOS2, IRF5, PTGS2), M2 macrophage (CD163, VSIG4, MS4A4A), CD8^+^ T cells (CD8A, CD8B), dendritic cells (CD1C, ITGAX), and CD4^+^ T cells (CD4) were also explored in LUAD. All these gene markers have been reported in previous published studies (28–35). The correlations in TIMER were adjusted by tumor purity with the left-most panel (36). The correlation modules were presented as the expression scatterplot for a pair of specified genes, as well as Spearman correlation and statistical significance. The *y*-axis represents the expression of GBE1, and the *x*-axis represents the expression of corresponding gene markers of immune cells. Gene expression levels were demonstrated by LOG2 RSEM.

### Statistical Analysis

The differences between categorical variables were analyzed by Chi-square test. The counting variables were expressed as mean ± standard deviation (SD), and the differences between counting variables were analyzed by Student’s *t*-test (number of variable = 2) or variance analysis (number of variable ≥2). The prognosis was estimated by Kaplan-Meier analysis with Log-rank test and Cox regression analysis. The strength of the correlation was determined using absolute values in TIMER, as shown below: very weak (0.00–0.19), weak (0.20–0.39), moderate (0.40–0.59), strong (0.60–0.79), and very strong (0.80–1.0) (30). Statistical analyses were processed using SPSS 23.0 software (SPSS, Inc., Chicago, IL, USA), and *p* < 0.05 was considered significant.

## Results

### The Expression Level of GBE1 Was Higher in LUAD Tissues

**Figure 1:**
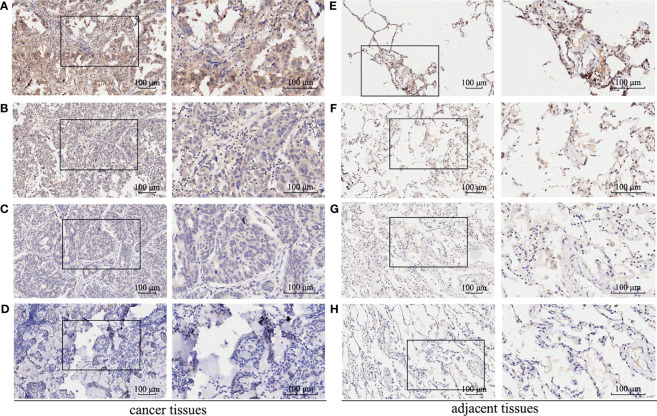
The immunohistochemistry staining of GBE1 in LUAD tissue samples and corresponding noncancer tissue samples. GBE1 protein levels were upregulated in most LUAD tissues compared with the corresponding noncancer tissues in the TMA‐IHC results. The representative TMA‐IHC images of different staining intensities of GBE1 were as follows **(A–H)**. **(A)** Strong intensity of GBE1 in LUAD tissue. **(B)** Moderate intensity of GBE1 in LUAD tissue. **(C)** Weak intensity of GBE1 in LUAD tissue. **(D)** Negative intensity of GBE1 in LUAD tissue. **(E)** Strong intensity of GBE1 in corresponding noncancer tissue. **(F)** Moderate intensity of GBE1 in tissue. **(G)** Weak intensity of GBE1 in corresponding noncancer tissue. **(H)** Negative intensity of GBE1 in corresponding noncancer tissue.

A total of 512 LUAD patients in TCGA database were selected, and the optimal cutoff value of GBE1 expression was 9.5. We firstly evaluated the differential GBE1 expression between 38 paired LUAD tissues and adjacent tissues. The results showed that GBE1 expression in LUAD tissues was higher than the adjacent tissues (**Table 1**). In TMA cohort, we analyzed the differential GBE1 expression between 81 paired LUAD tissues and adjacent tissues by IHC. The IHC results suggested that GBE1 was mainly expressed in the cytoplasm and overexpressed in LUAD tissues (**Table 1**). The different staining intensities of GBE1 are displayed in **Figure 1**.

**Table 1:** Differential expression of GBE1 in LUAD and normal tissues by utilizing the TCGA and TMA cohorts.

	TCGA cohort				TMA cohort			
	low expression	high expression	χ2	p	low expression	high expression	χ2	p
Normal tissues	33	5	13.41	<0.01	72	9	31.16	<0.01
LUAD tissues	18	20			39	42		

### High Expression of GBE1 Was Associated With Poor Prognosis in LUAD

**Figure 2:**
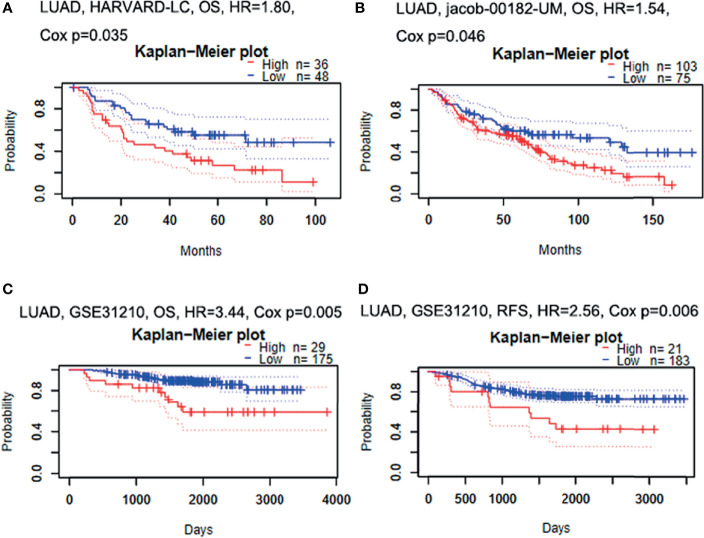
The relationship between GBE1 expression and prognosis in LUAD analyzed by PrognoScan database **(A–D)**. **(A)** In the HARVARD-LC cohort, high expression of GBE1 predicted a poor OS in LUAD (HR = 1.80, *p* = 0.035). **(B)** In the jacob-00182-UM cohort, high expression of GBE1 predicted a poor OS in LUAD (HR = 1.54, *p* = 0.046). **(C)** In the GSE31210 cohort, high expression of GBE1 predicted a poor OS in LUAD (HR = 3.44, *p* = 0.005). **(D)** In the GSE31210 cohort, high expression of GBE1 predicted a poor RFS in LUAD (HR = 2.56, *p* = 0.006). OS, overall survival; RFS, recurrence-free survival.

**Figure 3:**
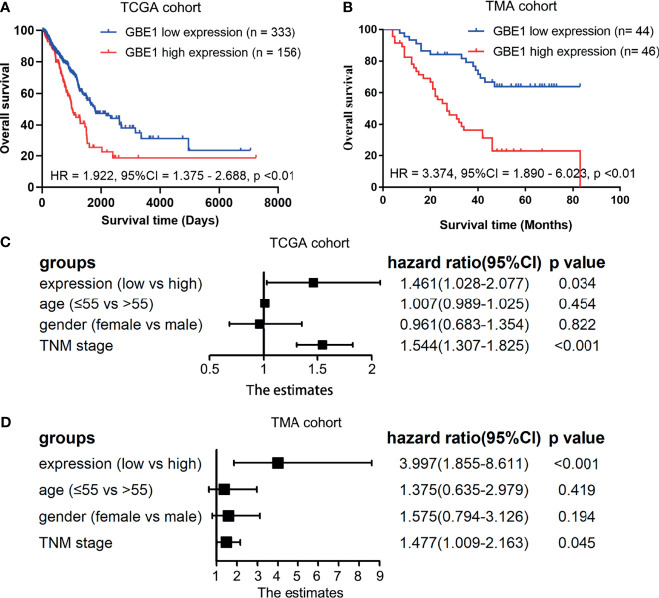
The correlations between GBE1 expression and prognosis in LUAD and the multivariable Cox regression analysis by utilizing the TCGA cohort and TMA cohort **(A–D)**. **(A)** The Kaplan-Meier survival curve comparing the high and low expression of GBE1 in TCGA cohort. **(B)** The Kaplan-Meier survival curve comparing the high and low expression of GBE1 in the TMA cohort. **(C)** The multivariable Cox regression analysis of different expression of GBE1 in TCGA cohort. **(D)** The multivariable Cox regression analysis of different expression of GBE1 in the TMA cohort.

Firstly, the correlation between GBE1 expression and survival was evaluated using the PrognoScan database. The results of three cohorts (HARVARD-LC, GSE31210, jacob-00182-UM) in PrognoScan showed that high expression of GBE1 had a poor prognosis in LUAD (**Figures 2A–D**). Furthermore, we also estimated the correlation between GBE1 expression and survival in TCGA cohort. The results revealed that 5-year survival rate of high-expression group of GBE1 in LUAD was significantly lower than that of low-expression group (low vs. high = 48.4% vs. 25.7%, **Figure 3A**). In addition, we also validated the prognostic value of GBE1 expression by using TMA-based IHC in 81 paired LUAD tissues and corresponding adjacent nontumor tissues. The result was consistent with that of PrognoScan database and TCGA cohort (low vs. high = 64.0% vs. 22.9%, **Figure 3B**).

To confirm whether GBE1 is an independent prognostic factor in LUAD patients, we performed Cox multivariable regression by using TCGA cohort and TMA cohort. The results showed that GBE1, age, gender, and TNM stage were independent prognostic factors (**Figures 3C, D**). Therefore, GBE1 has a potential to serve as a poor prognostic biomarker in LUAD patients.

### Correlation Between GBE1 Expression and Clinicopathological Characteristics of LUAD

**Figure 4:**
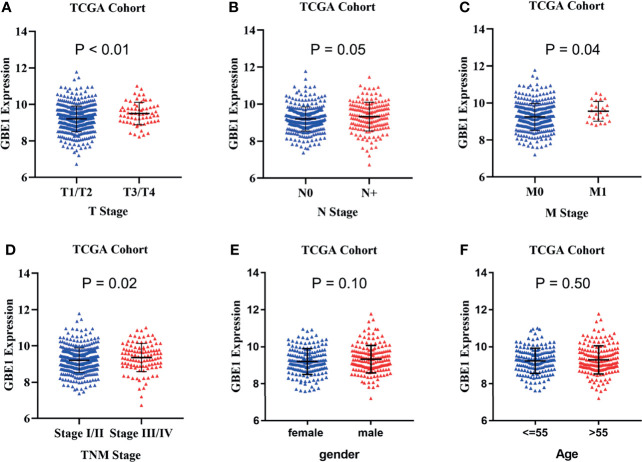
TThe correlation analysis between GBE1 expression and different clinical characteristics in LUAD via TCGA cohort. **(A)** The correlation analysis between GBE1 expression and T stage. **(B)** The correlation analysis between GBE1 expression and N stage. **(C)** The correlation analysis between GBE1 expression and M stage. **(D)** The correlation analysis between GBE1 expression and TNM stage. **(E)** The correlation analysis between GBE1 expression and gender. **(F)** The correlation analysis between GBE1 expression and age.

In TCGA cohort, we found that higher mRNA expression of GBE1 was correlated with advanced T classification (*p* < 0.01), N classification (*p* = 0.05), M classification (*p* = 0.04), and TNM stage (*p* = 0.02). However, there was no significant difference between GBE1 expression and age (*p* = 0.50) or gender (*p* = 0.10) (**Figures 4A–F**). We further explore the protein expression of GBE1 by utilizing the TMA cohort. The results showed that GBE1 was related with T classification (*p* = 0.004), N classification (*p* = 0.011), grade (*p* < 0.001), and TNM stage (*p* = 0.001) (**Table 2**). These results suggested that GBE1 may be associated with LUAD progression.

**Table 2:** Correlation between GBE1 expression and clinicopathological characteristics in TMA cohort.

	variables	GBE1 expression		total	χ2	p value
		low	high			
Age (years)					0.67	0.413
	≤ 55	17	14	31		
	> 55	27	32	59		
T stage					8.081	0.004
	T1/T2	34	24	58		
	T3/T4	4	15	19		
	null			3		
Sex					0	0.985
	Female	20	21	41		
	male	24	25	49		
TNM stage					10.54	0.001
	I/II	27	13	40		
	III/IV	5	16	21		
	null			29		
N stage					6.431	0.011
	N0	22	11	33		
	N+	13	23	36		
	null			21		
M stage						1.000*
	M0	43	45	88		
	M1	1	1	2		
ALK					0	1.000
	Negative	35	37	72		
	positive	5	5	10		
	null			8		
EGFR					0.813	0.367
	Negative	36	34	70		
	positive	8	12	20		
Grade					15.464	<0.001
	I	8	0	8		
	II	31	28	59		
	III	5	18	23		

*Fisher’s exact test.

### Relationship Between GBE1 Expression and Immune Marker Sets

**Figure 5:**
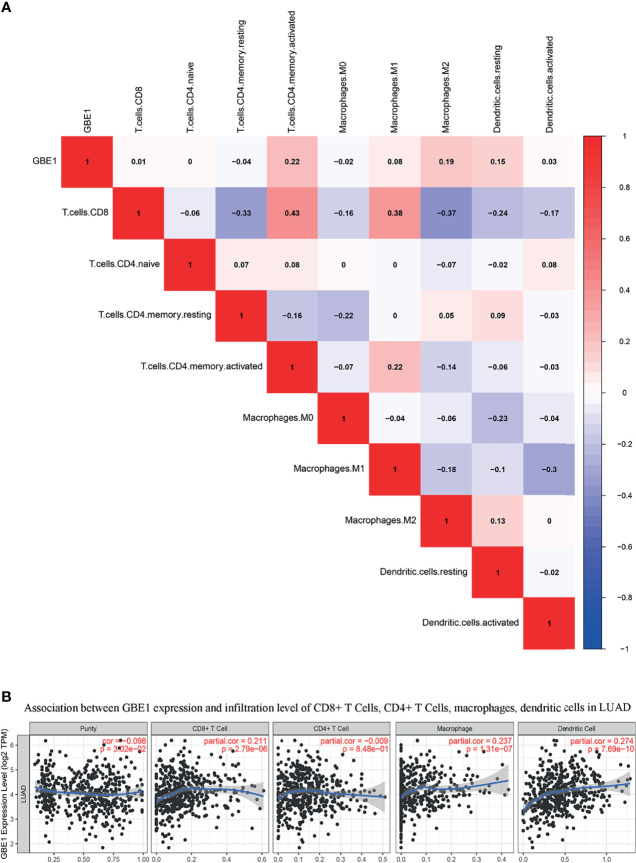
**(A)** The relationship between GBE1 expression and immune-infiltrating cells by utilizing CIBERSORT algorithm: GBE1 expression was positively correlated with M2 macrophage infiltration of M2 macrophages, CD4^+^ memory-activated T cells, and resting dendritic cells. **(B)** The relationship between GBE1 expression and immune-infiltrating cells by utilizing the TIMER database: GBE1 expression was positively correlated with macrophage infiltration, CD8^+^ T-cell infiltration, and dendritic cell infiltration.

**Figure 6:**
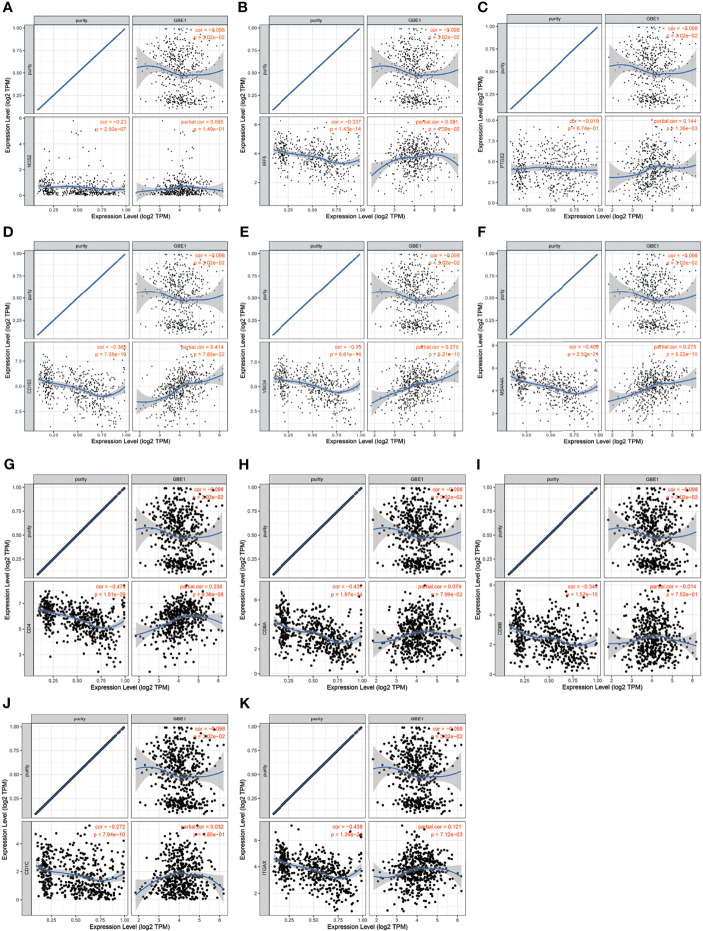
The correlation analysis between GBE1 and gene markers of tumor-associated immune cells in the TIMER database. **(A–C)** Gene markers of M1 macrophage; **(D–F)** gene markers of M2 macrophage; **(G)** gene marker of CD4+ T cell; **(H–I)** gene markers of CD8+ T cell; **(J–K)** gene markers of dendritic cell.

**Figure 7:**
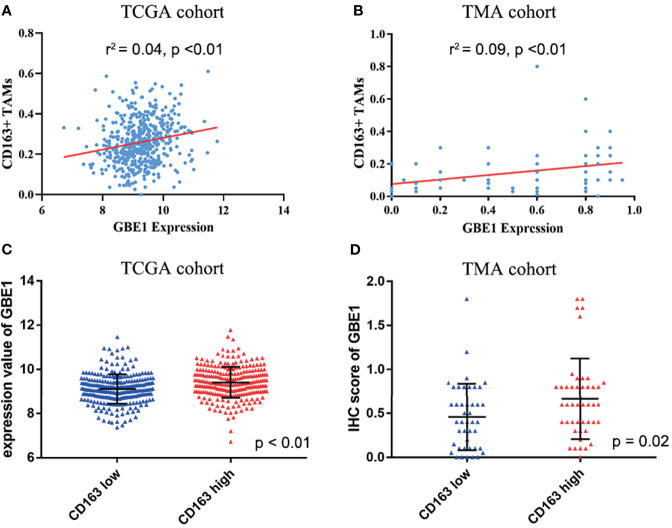
The relationship between GBE1 expression and M2 macrophage infiltrations in LUAD. **(A, B)** The correlations between GBE1 expression and CD163^+^ tumor-associated macrophage infiltration in TCGA and TMA cohorts (*r* ^2^ = 0.04, *p* < 0.01; *r* ^2^ = 0.09, *p* < 0.01). **(C)** The correlation between GBE1 expression and CD163 expression in TCGA cohort (*p* < 0.01). **(D)** The correlation between GBE1 expression and CD163 expression in the TMA cohort (*p* = 0.02).

**Figure 8:**
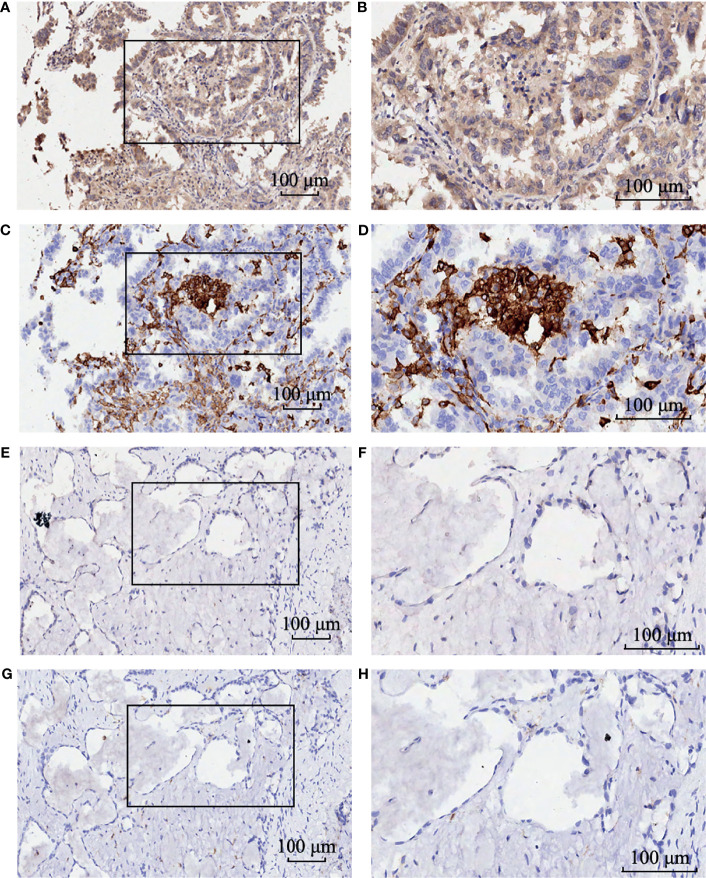
The immunohistochemistry staining of GBE1 and CD163 in the same LUAD tissue samples. Strong intensity of GBE1 in LUAD tissue **(A)** low magnification and **(B)** high magnification. Strong intensity of CD163 in LUAD tissue **(C)** low magnification and **(D)** high magnification. Weak intensity of GBE1 in LUAD tissue **(E)** low magnification and **(F)** high magnification. Weak intensity of CD163 in LUAD tissue **(G)** low magnification and **(H)** high magnification.

To investigate the relationship between GBE1 expression and immune-infiltrating cells, we used CIBERSORT algorithm to calculate the correlations between GBE1 mRNA expression and immune marker sets of various immune cells, including T cells and B cells, M1 and M2 macrophages, and dendritic cells (**Figure 5A**). The results revealed that GBE1 expression was positively correlated with infiltration of M2 macrophages, CD4^+^ memory-activated T cells, and resting dendritic cells. To further validate the relationships between GBE1 and infiltration of immune cells, we also analyzed the correlations between GBE1 expression and infiltration level of various immune cells by TIMER (**Figure 5B**). The results showed that GBE1 expression was positively correlated with macrophage infiltration, CD8^+^ T-cell infiltration, and dendritic cell infiltration. We also estimated the relationships between GBE1 and immune marker genes of immune cells, included M1 (NOS2, IRF5, PTGS2) macrophages, M2 (CD163, VSIG4, MS4A4A) macrophages, CD8^+^ T cells (CD8A, CD8B), dendritic cells (CD1C, ITGAX), and CD4^+^ T cells (CD4) in TIMER. The results showed that GBE1 exhibited positive association with CD163, VSIG4, and MS4A4A in LUAD (*r* = 0.414, *p* < 0.001; *r* = 0.275, *p* < 0.001; *r* = 0.275, *p* < 0.001). Moreover, GBE1 showed positive associations with markers of M1 macrophages and CD4, IRF5, PTGS2, and ITGAX in LUAD (**Figure 6**). Interestingly, CD163 mRNA expression was also positively correlated with GBE1 mRNA expression in TCGA cohort (**Figure 7A**). We further confirmed the relationship between GBE1 expression and CD163^+^ tumor-associated macrophage infiltration by using TMA-based IHC, and the IHC staining of the same tissues are shown in **Figures 8A–H**. The result revealed that GBE1 was strongly positively related to the CD163^+^ tumor-associated macrophage infiltration, which was consistent with the results of CIBERSORT algorithm, TIMER database, and TCGA cohort (**Figure 7B**). We dichotomized the infiltration level of CD163^+^ tumor-associated macrophages into low-infiltration group and high-infiltration group based on the median value. The result showed that the expression level of GBE1 was significantly lower in the low infiltration CD163 group when compared with high-infiltration group both in mRNA and protein levels (**Figures 7C, D**). These findings suggested that GBE1 may regulate macrophage polarization in LUAD. High expression of GBE1 may promote high infiltration level of CD163^+^ tumor-associated macrophages in LUAD.

## Discussion

In this study, we firstly found that GBE1 expression was higher in LUAD tissues when compared to normal tissues by utilizing TCGA database. The protein level of GBE1 in TMA cohort was also higher in LUAD tissues. Furthermore, we explored the prognostic value of GBE1 in LUAD by utilizing the HARVARD-LC cohort, Jacob-00182-UM cohort, GSE31210 cohort, and TCGA cohort. All these results showed that the LUAD patients with higher expression of GBE1 had a poorer prognosis than patients with lower GBE1 expression. The prognostic analyses from the TMA cohort also showed that higher GBE1 expression indicated a poor prognosis in LUAD patients. To confirm whether GBE1 is an independent prognostic factor, we performed a multivariate Cox regression and the results showed that GBE1 was associated with prognosis independently. In addition, the expressions of GBE1 in protein and mRNA levels were positively associated with T classification, N classification, pathological grade, and TNM stage in LUAD patients. To our knowledge, this is the first study to explore the association between GBE1 expression and clinical characteristics in LUAD patients. Therefore, our study shed light on the role of GBE1 in tumor progression and its potential to be a prognostic biomarker.

Previous study indicated that GBE1 expression was upregulated in acute myelocytic leukemia and the level of GBE1 was associated with the efficacy of anti-PD1 treatment in melanoma patients (37, 38). Although GBE1 has not been extensively studied in LUAD, it was reported that GBE1 knockdown could increase the expressions of CCL5 and CXCL10 in A549 cells through the STING/IFN-I pathway, then promoting the recruitment of CD8^+^ T lymphocytes and PD-L1 overexpression, which may improve prognosis and the efficacy of anti-PD-L1 treatment (6). In addition, GBE1 overexpression could trigger the conversion of anaerobic glycolysis and enhance glucose uptake by inhibiting expression of FBP1 and improving HIF1α expression, then promote LUAD progression (5).

The roles of TAMs in tumor progression have been noticed (39). Previous studies have showed that TAMs were correlated with prognosis in colorectal cancer, breast cancer, etc. (40–43). TAMs also play important roles in cancer development and progression. Under the regulation of microenvironmental signals, macrophages would polarize into classically activated macrophages (M1) and alternatively activated macrophages (M2) (44). Proinflammatory M1 macrophages could phagocytose cancer cells, while anti-inflammatory CD163^+^ tumor-associated macrophages could promote tumor growth and invasion (45). Previous studies had reported that M2 macrophage may promote LUAD progression and was associated with the survival of NSCLC patients (46, 47).

In this study, we explored the correlation between GBE1 and immune cell infiltration by utilizing CIBERSORT algorithm. The result of the CIBERSORT algorithm revealed that GBE1 expression was significantly correlated with infiltration of CD163^+^ tumor-associated macrophages. Furthermore, the correlations between GBE1 and the immune cells gene markers indicated that GBE1 exhibited moderate association with CD163. We further used TMA-based IHC to confirm the positive relationship between GBE1 expression level and CD163^+^ tumor-associated macrophage infiltration. Our results implied that expression of GBE1 may be related with CD163^+^ tumor-associated macrophage infiltration in LUAD. The expression level of GBE1 was associated with prognosis and CD163^+^ tumor-associated macrophage infiltration in LUAD, suggesting its potential utility as a prognostic biomarker and immune-related therapeutic target for LUAD patients.

We must acknowledge that there are some limitations worth noting in our study. First, our study only found that GBE1 may be associated with LUAD progression and CD163^+^ TAM infiltration, but we did not explore the exact mechanism. In future study, we would construct overexpressed GBE1 LUAD cell line and silence-GBE1 LUAD cell line to carry on further functional experiments and mechanism experiments. We will explore how GBE1 plays the role in macrophage differentiation and polarization and whether the expression of GBE1 could influence the secretion of cytokines and/or metabolites that are involved in M2 polarization and in macrophages recruitment. Furthermore, we will explore whether LUAD cells could release GBE1 and function in the extracellular space. Second, we only used 90 LUAD tissues to verify the effect of GBE1 on prognosis and the relationship between GBE1 and CD163^+^ TAMs infiltration. We will enlarge the sample size to further confirm these results and explore whether GBE1 could affect infiltration of other immune cells. The underlying molecular mechanisms of GBE1 in LUAD are also worth being further explored.

In conclusion, high expression of GBE1 is associated with poor prognosis and could improve immune infiltration levels of CD163^+^ TAMs in LUAD. GBE1 may play an important role in CD163^+^ TAM infiltration and has a potential to be a prognosis biomarker in patients with LUAD.

## Data Availability Statement

The original contributions presented in the study are included in the article/supplementary material. Further inquiries can be directed to the corresponding author.

## Ethics Statement

The studies involving human participants were reviewed and approved by the Ethics Committee of the National Cancer Center/Cancer Hospital, Chinese Academy of Medical Sciences and Peking Union Medical College (IRB Approval No. NCC2019C-167). The patients/participants provided their written informed consent to participate in this study.

## Author Contributions

YiL: conceptualization (lead), formal analysis (lead), and methodology (lead). YaL: methodology (equal), project administration (equal), supervision (equal), validation (equal), and writing—original draft (lead). ML: validation (equal) and writing—original draft (supporting). MD: data curation (equal), resources (equal), and writing—original draft (supporting). ZL: validation (equal) and writing—original draft (supporting). XL: supervision (supporting). XM: supervision (supporting). BZ: supervision (supporting). YG: funding acquisition (lead), project administration (lead), and writing—review and editing (lead).

## Funding

This work was supported by the National Key R&D Program of China (Grant Nos. 2020YFE02022200).

## Conflict of Interest

The authors declare that the research was conducted in the absence of any commercial or financial relationships that could be construed as a potential conflict of interest.

## Publisher’s Note

All claims expressed in this article are solely those of the authors and do not necessarily represent those of their affiliated organizations, or those of the publisher, the editors and the reviewers. Any product that may be evaluated in this article, or claim that may be made by its manufacturer, is not guaranteed or endorsed by the publisher.

## Abbreviations

GBE1, glycogen branching enzyme; LUAD, lung adenocarcinoma; TCGA, The Cancer Genome Atlas; TIMER, Tumor Immune Estimation Resource; TMA, tissue microarray; OS, overall survival; TAMs, tumor-associated macrophages; Tregs, regulatory T cells; NSCLC, nonsmall-cell lung cancer; IHC, immunohistochemistry; AJCC, American Joint Committee on Cancer; HR, hazard ratio; SD, standard deviation; RFS, relapse-free survival.
